# Comparison of impact and cost-effectiveness of rotavirus supplementary and routine immunization in a complex humanitarian emergency, Somali case study

**DOI:** 10.1186/s13031-015-0032-y

**Published:** 2015-02-09

**Authors:** Lisa M Gargano, Jacqueline E Tate, Umesh D Parashar, Saad B Omer, Susan T Cookson

**Affiliations:** Center for Global Health, Centers for Disease Control and Prevention, 4770 Buford Highway, Atlanta, GA 30341 USA; National Center for Immunization and Respiratory Diseases, Centers for Disease Control and Prevention, Atlanta, GA USA; Rollins School of Public Health, Emory University, Atlanta, GA USA

**Keywords:** Rotavirus vaccine, Humanitarian emergency, Somalia, Cost-effectiveness, Routine immunization

## Abstract

**Background:**

A humanitarian emergency involves a complete breakdown of authority that often disrupts routine health care delivery, including immunization. Diarrheal diseases are a principal cause of morbidity and mortality among children during humanitarian emergencies. The objective of this study was to assess if vaccination against rotavirus, the most common cause of severe diarrhea among children, either as an addition to routine immunization program (RI) or supplemental immunization activity (SIA) would be cost-effective during a humanitarian emergency to decrease diarrhea morbidity and mortality, using Somalia as a case study.

**Methods:**

An impact and cost-effectiveness analysis was performed comparing no vaccine; two-dose rotavirus SIA and two-dose of RI for the 424,592 births in the 2012 Somali cohort. The main summary measure was the incremental cost per disability-adjusted life-year (DALY) averted. Univariate sensitivity analysis examined the extent to which the uncertainty in the variables affected estimates.

**Results:**

If introduced in Somalia, a full-series rotavirus RI and SIA would save 908 and 359 lives, respectively, and save US$63,793 and US$25,246 in direct medical costs, respectively. The cost of a RI strategy would be US$309,458. Because of the high operational costs, a SIA strategy would cost US$715,713. US$5.30 per DALY would be averted for RI and US$37.62 per DALY averted for SIA. Variables that most substantially influenced the cost-effectiveness for both RI and SIA were vaccine program costs, mortality rate, and vaccine effectiveness against death.

**Conclusions:**

Based on our model, rotavirus vaccination appears to be a cost-effective intervention as either RI or SIA, as defined by the World Health Organization as one to three times the per capita Gross Domestic Product (Somalia $112 in 2011). RI would have greater health impact and is more cost effective than SIA, assuming feasibility of reaching the target population. However, given the lack of infrastructure, whether RI is realistic in this setting remains unanswered, and alternative approaches like SIA should be further examined.

## Introduction

A complex humanitarian emergency involves a breakdown of authority that goes beyond the mandate or response capacity of any single country or United Nations agency [1]. This leads to the creation of refugees (those who leave their country of origin) and internally displaced persons (IDPs) (those who are displaced but remain in their country). At the end of 2012, there were 45.2 million people worldwide forcibly displaced due to conflict and persecution [2]. On average, 13% of all persons of concern (refugees, IDPs, stateless persons, and those recently returned home) were children under the age of five years [2]. Humanitarian emergencies, regardless of timing, have a number of common risk factors for communicable diseases including mass population movement and resettlement in temporary locations, overcrowding or even absence of shelter, economic and environmental degradation, impoverishment, scarcity of safe water, poor sanitation and waste management, poor nutritional status as a result of food shortages, and poor access to health care. These risk factors are inextricably linked to excess risk of morbidity and mortality from waterborne and vaccine preventable diseases, the reduction of which is the aim of public health interventions during crises. These emergencies can be sudden or of a more protracted with a longer term effect as it currently being seen in Somalia and Syria, both of which are seeing ongoing insecurity, continued displacement of people, and lack of sound governance impacting the population’s health and wellbeing.

Infectious diseases continue to cause high levels of morbidity and mortality during a humanitarian emergency. Measles in children has been shown repeatedly to be a major, and often the most important, cause of death among refugee and displaced children [3]. However through supplemental immunization campaigns, mortality from this cause has been greatly reduced [3]. Today, diarrheal diseases are a principal cause of morbidity and mortality in humanitarian emergencies [4].

Worldwide, rotavirus is the most common cause of severe diarrhea among infants and young children [5,6]. Where appropriate resources are available, rotavirus is usually an easily managed disease of childhood, but globally the World Health Organization (WHO) estimates that 453,000 children under the age of five years died of rotavirus in 2008 [7,8] and almost two million more become severely ill [9]. In developing countries, the majority of severe rotavirus disease occurs during the first year of life [10].

In 2006, two vaccines against rotavirus infection were shown to be safe and effective in children: two-dose Rotarix by GlaxoSmithKline™ and three-dose RotaTeq by Merck™ [11,12]. Both are administered orally and contain attenuated live virus [13]. As of January 2014, 53 countries have introduced rotavirus vaccines through their national immunization programs, including 20 low-income countries eligible for vaccine introduction support from Gavi, the Vaccine Alliance (formerly the Global Alliance for Vaccines and Immunizations) [14]. The incidence and severity of rotavirus infections has declined significantly in countries that have acted on WHO recommendations and introduced the rotavirus vaccine into their routine immunization programs [15]. In 2013, WHO published a position paper recommending the use of rotavirus vaccines in all national immunization programs, particularly in south and south-eastern Asia and sub-Saharan Africa [16]. Somalia has not introduced rotavirus vaccination into its routine immunization program.

A 1991 military coup in Somalia, followed by years of civil war, devastated much of the country’s infrastructure, including its healthcare system [17]. Multiple attempts to restore a stable national government have failed and much of Somalia remains in protracted conflict, with large populations of IDPs having inadequate access to health and social services, especially in the two southern zones [18]. In 2012, Somalia was the second largest country of origin for refugees, with more than 1.1 million people fleeing due to conflict, violence, drought, and famine [2]. Indicators of the collapse of the health system include child and maternal mortality rates that rank among the highest globally [19] and record low levels of routine child immunization coverage, which have remained at these levels for the past 20 years [20], from 19% three-dose diphtheria, tetanus, and pertussis (DTP3) coverage in 1997 to 34% in 2013 [21,22]. Somalia’s routine immunization services are heavily supported by UNICEF and partner agencies. However, security problems and inadequate resources hamper coverage of these services [23]. As a result, much vaccination is accomplished through supplemental immunization activities (SIAs), each occurring over a few-day period at the regional level and larger areas [24].

An analysis of the cost-effectiveness of rotavirus vaccine found that in 68 of the 72 (94%) Gavi-eligible countries the vaccine was considered cost-effective [25]. Although studies exist, specifically with new vaccines, there have been no analysis published during a humanitarian emergency using a SIA, despite the establishment of the WHO/ Strategic Advisory Group of Experts (SAGE) on Immunization framework for decision-making of vaccination in acute humanitarian emergencies [26].

The objective of this study was to determine the potential health impact and cost-effectiveness of adding rotavirus vaccine to the routine immunization program (RI) and of a national supplemental rotavirus vaccination campaign in Somalia, a country that has experienced a protracted humanitarian emergency. This analysis was conducted from the publicly funded healthcare system perspective to aid governments and donor organizations in decisions about whether to introduce rotavirus vaccination during a humanitarian emergency.

## Methods

### Overview of model

Disease-specific, decision-tree models were developed to estimate the impact and cost-effectiveness of rotavirus vaccination in both a national routine vaccination program and a national supplemental rotavirus immunization activity (SIA) by comparing the cost and burden of disease with and without such administration. We examined Rotarix since it requires fewer total doses (2 versus 3 for RotaTeq) and may be more amenable to use in emergency. The model was created for use in Somalia and estimated health outcomes and direct medical costs associated with rotavirus disease for the 2012 Somalia annual birth cohort of 424,594 infants followed for one year [27]. For SIAs, at any one time, we assumed that 50% of the birth cohort would be eligible to receive two-doses of the vaccine given in a hypothetical 26-week dosing window (between 6 and 32 weeks of age). We also adjusted for those children who would have died from rotavirus disease before vaccination occurred among those receiving SIA by calculating that 38% of deaths would have occurred prior to vaccination, leaving 62% of deaths potentially prevented during the 26-week age window assuming equal age distribution in the vaccine eligible window [28]. For RI, we assumed rotavirus vaccine would be given at the recommended ages (6 and 10 weeks) for the first two doses of vaccines in the routine immunization program. Variables considered in the analysis are listed in Table 1. The model inputs include diarrhea and rotavirus incidence; vaccine coverage, effectiveness, and cost (administrative and price of vaccine); medical care service costs and proportion utilizing services; and mortality rate. We did not consider direct non-medical costs, such as transportation costs or indirect costs, such as time lost to parents. We chose to estimate the burden of disease during the first year of life instead of during the first five years because of the mobility of the population and because in other African countries, over 82% of rotavirus infections occur among children under one year of age [29].

**Table 1:** **Principle base-case values, sensitivity ranges, and references for model inputs for estimating cost-effectiveness of a rotavirus vaccination campaign in Somalia**

**Parameter**	**Base-case estimate**	**Sensitivity range**	**References**
**Epidemiologic**			
2012 Birth cohort	424,594	NA	[27]
Diarrhea disease 2-week incidence, %	21	15.75-26.25 (+/− 25%)	[30]
Diarrhea cases, severe^a^, %	6	NA	[31]
Diarrhea cases, moderate^a^, %	42	NA	[31]
Diarrhea cases, mild^a^, %	52	NA	[31]
Rotavirus incidence severe^b^, %	48	NA	[29,32]
Rotavirus incidence moderate^c^, %	35	NA	[7]
Rotavirus incidence mild/subclinical^d^, %	17	NA	[7]
Receive care, %	25	18.75-31.25 (+/−25%)	[30]
Mortality associated with Rotavirus (per 1,000)	9.1	4.55-13.65 (+/− 50%)	[29,33]
**Clinical**			
Proportion eligible for vaccination at SIA, %	50	NA	Assumption
SIA coverage, %	60	45-75 (+/−25%)	[34-37]
RI coverage, %	47	35.25-58.75 (+/− 25%)	[38]
Vaccine effectiveness, %			
Death	50	37.5-62.5 (+/−25%)	[33]
Severe	50	37.5-62.5 (+/−25%)	[33]
Moderate	40	30-50 (+/−25%)	[33]
Mild/subclinical	30	22.5-37.5 (+/−25%)	Assumption
Length of hospital stay severe, days	4	NA	[32,39]
Length of hospital stay moderate, days	2	NA	[32]
**Economic, US$**			
Gavi price per dose	0.15	NA	[40]
SIA operational cost per child	3.00	1.00-4.00	Personal communication
RI operational costs per child	0.34	0.05-1.00	[33,39]
Cost per day of care	1.01	NA	[41]

^a^of all diarrhea.

^b^of severe diarrhea cases.

^c^of moderate diarrhea cases.

^d^of mild diarrhea cases.

### Model inputs

#### Disease burden

UNICEF’s Somalia multiple indicator cluster survey 3 (MICS3) conducted in 2005–2006 estimated the two-week diarrhea incidence for children aged under five years to be 21% [30]. We determined rates of severe, moderate, and mild all-cause diarrhea among children less than 5 years of age from published estimates [31]. Since this analysis is focused on children under one year of age and during an emergency with malnutrition and poor hygiene, we decreased the proportion of mild diarrhea cases by 20%, from 64.8% to 52%, and increased the proportion of moderate by 20%, from 34.7% to 42%. We then adjusted severe diarrhea cases accordingly, from 0.5% to 6%, to total 100%. To determine the rate of severe rotavirus diarrhea, severe diarrhea was multiplied by the proportion of diarrhea due to rotavirus (48%) detected through hospital surveillance among Malawi children under one year of age, since no Somalia specific data were available and Malawi is the closest comparable country from which data were available [29,32]. We assumed that rotavirus detection rates decreased with decreasing severity of disease by approximately 25% for each severity decrease, as previously described [7].

#### Mortality

To estimate the rotavirus mortality rate, we assumed that proportion of diarrheal hospitalizations due to rotavirus approximates the proportion of diarrheal deaths due to rotavirus [33,42]. To determine the diarrheal mortality rate, the total under-one years of age mortality rate in Somalia (103.72 per 1,000 live births) was multiplied by the estimated proportion of all deaths attributable to diarrhea [43]. There are no good estimates for deaths due to diarrhea in Somalia so we used published estimates for under-5 years of age in Mali (18.3%) [44]. To determine the rotavirus mortality rate, this rate was multiplied by the percent of severe diarrhea attributable to rotavirus among children under one year old (48%) [29,32]. Therefore, the rotavirus-associated mortality among those less than one year of age was found to be 9.1 per 1,000 (Table 1).

#### Healthcare utilization

We assumed the proportion of children who would receive care would be equivalent to Somalia’s MICS3 report that 25% of children aged under five years received some form of oral rehydration therapy for diarrhea [30]. Because of the large number of IDPs with inadequate access to health services, especially in the two southern zones [18], for severity of illness we did not differentiate level, but only length of care. Among those children receiving care, based on published reports we assumed children with severe cases of rotavirus would have four days of care and moderate cases would have two days of care [32,39].

#### Costs

Cost estimates were obtained from several sources. We used the Gavi-subsidized vaccine price for the Rotarix vaccine of $0.15 per dose [45]. The operational costs were estimated by UNICEF at $3.00 per child per immunization campaign, which was equal to the cost of measles vaccination per child in Somalia (Heather Papowitz, personal communication, August 14, 2013). No data were available for RI costs in Somalia so we assumed it would be US$0.34 similar to other published reports from African countries [32,33]. When determining the number of doses needed for a RI, a wastage rate of 10% was used [33]. We used standardized WHO-CHOICE (CHOosing Interventions that are Cost Effective) to estimate the per diem costs of a health care visit [41]. Somalia estimates were not available so we used the average of Ethiopia and Kenya, two neighboring countries where the majority of Somali refugees seek asylum. The cost of care was US$1.01 per day. No discounting was taken into account because the analytic horizon was only one year.

#### Vaccine program coverage and effectiveness

For SIA base-case estimates and lower and upper limit of vaccination coverage, we used several data sources for vaccination coverage following measles supplemental immunization activities from Darfur, Sudan, Afghanistan, and refugee camps in Kenya [34-37]. Vaccine coverage for RI was estimated to be 47%, the median between the 2012 RI coverage of DTP1 (52%) and DTP3 (42%) obtained from the WHO vaccine-preventable diseases monitoring system [46].

For both SIA and RI, rotavirus vaccine provides heterotypic immunity with better protection against severe rotavirus disease [33]. Efficacy against severe rotavirus-associated disease was assumed to be 50% for full-series based on clinical trial data from Africa [47]. No specific data of rotavirus efficacy against moderate cases exist from a country comparable to Somalia; therefore, we assumed effectiveness against moderate cases of rotavirus to be 20% lower than the estimated effectiveness against severe disease [33]. Given that vaccine effectiveness against office visits was lower than effectiveness against hospitalizations in clinical trials in developed countries, we assumed that vaccine effectiveness against mild rotavirus disease was 40% lower than that for severe disease.

### Data analysis

#### Decision analysis model

The costs and benefits of implementing a rotavirus immunization campaign during a humanitarian emergency were compared to no vaccination using Excel 2010 (Redmond, WA). The model was analyzed under base case scenario to determine the costs of different options: no vaccination, full-series of Rotarix vaccine via RI or full-series of Rotarix vaccine via SIA. A one-way sensitivity analysis was conducted to evaluate the impact that changes in values of several parameters would have on the main model outcome, incremental cost-effectiveness ratio.

#### Cost-effectiveness

The main summary measure for the incremental cost-effectiveness ratio (ICER) was cost per disability-adjusted life-years (DALY) averted, expressed in 2013 US dollars. DALYs were estimated using the WHO life expectancy data with age weighting. The disability weight for severe diarrhea in children under five years of age from “The 2010 Global Burden of Disease” was used to calculate years lost due to disability [48], with the mean duration of diarrhea of three days [33]. The life expectancy tables used were last produced in 2010 [49]. Survivors of both severe and moderate infections are not known to have any long-term disability resulting from infection [32]; therefore, the DALYs were almost entirely based on the years of life lost [50].

#### Sensitivity analysis

Starting from the base case scenario, univariate sensitivity analyses were carried out to examine the extent to which the uncertainty in the variables affected our estimates. We conducted sensitivity analyses for vaccination coverage for both SIA and RI [51]. Vaccine effectiveness of any oral antigen is dependent on many factors, such as vaccine potency at time of administration (cold chain maintenance), presence of diarrhea, nutritional status, and receipt of vitamin A. Therefore, sensitivity analyses were performed on this variable as well [52]. Healthcare utilization varies depending on presence of facilities and mobility of the target population, in part because of insurgents limiting mobility and access to facilities. In addition, access to clean water and sanitation can affect disease burden. Since access to these services is uncertain during a humanitarian emergency, we performed sensitivity analysis on use of healthcare systems, diarrheal disease burden, and rotavirus-associated mortality rates (Table 1).

#### Additional assumptions

Several additional assumptions were made. First, we assumed that the proportion of diarrhea due to rotavirus was similar between the general population in developing countries and the humanitarian emergency-affected population. Second, baseline rotavirus vaccine coverage was assumed to be zero. Third, we assumed that all vaccinated children were vaccinated at the appropriate age or age range for SIA and correct time interval between doses.

## Results

### Disease and economic burden of rotavirus disease in the absence of vaccination program

In 2012, we estimated that among children less than 1 year of age, rotavirus diarrheal illnesses resulted in 3,864 deaths, 606,917 cases, and 100,961 children receiving care (Table 2). The total annual cost of the burden of rotavirus disease described above would be US$322,618 (Table 2).

**Table 2:** **Rotavirus-related events or costs with and without rotavirus vaccine and averted outcomes with vaccination, (lower limit, upper limit)**

	**Without vaccine**	**With RI**	**Averted by vaccine**	**% reduction**	**With SIA**	**Averted by vaccine**	**% reduction**
**No. of events**							
Deaths	3,864	2,956 (1,478-4,434)	908 (454–1,362)	23.5	3,504 (1,752-5,257)	359 (180–539)	9.3
Cases	606,917	499,251 (374,438-624,064)	107,665 (80,749-134,582)	17.7	564,309 (423,232-705,386)	42,608 (31,956-53,260)	7.0
Receive care	100,961	81,203 (60,902-101,504)	19,758 (14,818-24,697)	19.6	93,142 (69,857-116,428)	7,819 (5,864-9,774)	7.7
Number of DALYs	197,683	151,308 (76,236-226,381)	46,375 (23,313-69,437)	23.5	179,331 (90,323-268,338)	18,353 (9,226-27,479)	9.3
DALYs averted per 1000 children			109 (55–163)			43 (22–65)	
**Medical treatment costs (US$)**	322,618	258,825 (194,119-323,532)	63,793 (36,067-79,741)	19.8	297,372 (223,029-371,716)	25,246 (18,934-31,557)	7.8
**Vaccination program costs (US$)**	--	309,458 (126,309-726,279)	--	--	715,713 (261,292-942,923)	--	--
**Net costs (medical plus vaccination campaign, US$)**	--	568,283 (385,135-985,104)	--	--	1,013,085 (558,664-1,240,295)	--	--

### Impact of vaccination

For a rotavirus RI, the number of deaths would be reduced to 2,956, a 23.5% reduction from no vaccine or 908 deaths averted and would prevent the loss of 109 DALYs per 1000 children. The total number of cases would be reduced to 499,251, a 17.7% reduction or 107,665 cases averted. The number of children receiving care would be 81,203, a 19.6% reduction or 19,758 children receiving care averted (Table 2). We estimated that the introduction of a full-series of rotavirus vaccine in a SIA would potentially decrease the number of deaths to 3,504, a 9.3% reduction from no vaccine or 359 deaths averted and would prevent the loss of 43 DALYs per 1000 children. The total number of cases would be reduced to 564,309, a 7.0% reduction or 42,608 cases averted. The number of children receiving care would be 93,142, a reduction of 7.7% or 7,819 children receiving care averted (Table 2).

Introduction of rotavirus into the RI would cost US$309,458. Healthcare costs would decrease by 19.8% or US$63,793 to US$258,825 (Table 2). A full-series rotavirus SIA program would cost US$715,713. Healthcare costs would decrease by 7.8% or US$25,246 to $297,372 (Table 2).

### Cost-effectiveness

The ICER for rotavirus given in a RI was US$5.30 per DALY averted (Table 3). The base-case scenario ICER for a full-series SIA strategy was US$37.62 per DALY averted (Table 3). We also examined cost per case averted and cost per death averted. A rotavirus RI is US$2.81 per case averted and US$270.56 per death averted (Table 3). A full-series SIA strategy is US$16.20 per case averted and US$1,921.58 per death averted (Table 3).

**Table 3:** **Cost-effectiveness of rotavirus vaccine, (lower limit, upper limit)**

**Incremental cost-effectiveness ratio (US$)**	**RI value**	**SIA value**
Cost per DALY averted	5.30 (1.35-14.28)	37.62 (12.86-74.93)
Cost per case averted	2.81 (0.58-6.15)	16.20 (5.54-21.54)
Cost per death averted	270.56 (68.85-729.62)	1,921.52 (656.90-2,553.83)

### Sensitivity analysis

**Figure 1:**
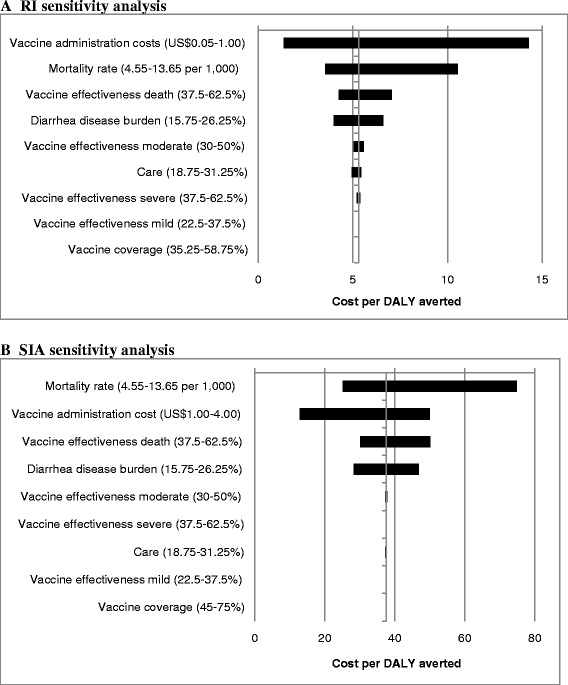
**Tornado diagrams of univariate sensitivity analysis of rotavirus vaccination campaign. (A)** Sensitivity analysis for RI and **(B)** Sensitivity analysis for SIA.

Figure 1 shows a tornado analysis of sensitivity. The variables that most affected the ICER for both full-series RI and SIA of rotavirus vaccine were: (1) vaccine program costs, (2) rotavirus-associated mortality rate, and (3) vaccine effectiveness against death.

For the RI, when program costs varied from US$0.05 to US$1.00 the resulting ICER ranged from US$1.35 to US$14.28 per DALY averted, respectively (Figure 1). When the mortality was varied from 4.55 per 1,000 to 13.65 per 1,000, the ICER ranged from US$10.54 to US$3.54 per DALY averted, respectively. When vaccine effectiveness against death varied from 37.5% to 62.5%, the resulting ICER ranged from US$7.05 to US$4.24 per DALY averted, respectively (Figure 1). The net cost (medical plus vaccine program costs) of a RI strategy would be between US$385,135 and US$985,104 and avert up to 1,362 deaths and 134,582 cases (Table 2).

For SIA, when program costs varied from US$1.00 to US$4.00 the resulting ICER ranged from US$12.86 to US$50.00 per DALY averted, respectively (Figure 1). When the mortality rate ranged from 4.55 per 1,000 to 13.65 per 1,000, the ICER ranged from US$74.93 to US$25.09 per DALY averted for SIA. When vaccine effectiveness against death varied from 37.5% to 62.5%, the resulting ICER for SIA ranged from US$50.07 to US$30.13 per DALY averted (Figure 1). The resulting net cost (medical plus vaccine campaign costs) of a SIA strategy would be between US$558,664 and US$1,240,295 and avert up to 539 deaths and 53,260 cases (Table 2).

## Discussion

This analysis found that administering rotavirus vaccine in a routine immunization strategy would have a greater health impact and better cost-effectiveness than SIA. While rotavirus immunization appears to be more cost-effective if given in a RI it is important to remember that because of the lack of infrastructure and trained workforce in Somalia; only about 40% of vaccines administered are given within the RI infrastructure (A. Kebede, WHO-Somalia, personal communication, March 5, 2014). This lack of infrastructure would probably result in additional costs to RI with the introduction of rotavirus vaccine, which were not taken into consideration. Therefore, the cost-effectiveness of RI may be an over-estimate given the need for improvements in cold chain and logistics with the addition of another antigen. This study serves to show the cost-effectiveness of rotavirus vaccine in both RI and SIA. When RI does not function during humanitarian emergencies, SIAs may be conducted to decrease disease burden and mortality.

According to WHO, a cost-effective intervention is when the ICER is less than three times the per capita GDP and *very* cost-effective intervention when the ICER is less than one times the per capita GDP (US$112 in 2011) [53,54]. The present study shows that vaccinating against rotavirus during a humanitarian emergency may be *very* cost-effective regardless of the strategy. Rotavirus vaccination may also be cost-effective in the neighboring refugee camps of Ethiopia and Kenya, where the majority of Somali refugees fled during the 2011 famine and subsequently [2], assuming the cost of vaccination and medical costs are not too much greater. In addition, while long-term efficacy of a single dose of rotavirus vaccine is not known, in practice some children may only receive one-dose. A one-dose SIA using 75% vaccine coverage, a rate achievable in other SIAs [34-37], and a vaccine effectiveness of 25% against death and severe disease, 20% against moderate disease, and 15% against mild/subclinical disease, which is 50% of that of a full course [33,55] would still reduce morbidity and mortality and be cost-effective at US$37.62 per DALY averted. While there are certain scenarios presented such as low diarrheal disease incidence and low mortality rate where SIA is no longer cost-effective, the reality of the situation during a humanitarian emergency makes these conditions unlikely.

Findings from these analyses agree with findings from previous cost-effectiveness analysis for rotavirus vaccine introduction into developing countries, including those countries that have experienced humanitarian emergencies, such as Uganda, Democratic Republic of Congo, and Afghanistan, that rotavirus vaccine would be cost-effective [25,33,50,56,57]. Like other studies, variables most influencing the ICER in the sensitivity analysis were program costs and mortality rate [32,33]. Changes in vaccine coverage did not have a substantial impact on cost-effectiveness estimates in the sensitivity analysis, which is similar to other published findings [33,39]. The high operational costs of SIAs make it substantially more expensive than if delivered within the RI and greatly decreases the cost-effectiveness of the SIA program compared to the RI. However, if it was given at the time of measles SIA, this administrative cost could be greatly reduced.

Recent findings from the Global Enteric Multicenter Study (GEMS) reinforces the finding that rotavirus is a major cause of moderate to severe diarrhea among children less than five years old [6]. Exacerbation of endemic rotavirus disease pattern during an emergency can be due to intense transmission and/or increase in case-fatality rate as a result of malnutrition, lack of health infrastructure, and low access to existing health services [26,58]. For those surviving, these episodes of diarrhea can also substantially affect physical growth, further justifying the use of rotavirus immunization alongside other diarrhea prevention strategies [59].

According to Moodley, *et al.*, finding affordable measures, including immunization, to prevent unnecessary illness and death in a humanitarian emergency is both beneficial and cost-effective. One of the primary measures to prevent unnecessary illness and death from diarrheal diseases in emergency settings is through hygiene promotion activities, such as improving hand washing practices. Although these practices should continue, in 2005–2006, Somali mothers had poor hand washing practices; only 45.4% reported washing hands after cleaning babies bottoms and 34.4% before feeding babies [30]. Providing an immunization intervention for a major cause of diarrheal morbidity and mortality among infants in humanitarian emergencies becomes a humanitarian consideration in addition to a cost-benefit consideration. For rotavirus, the WHO/SAGE decision-making factors for vaccine deployment appear to have all been met: the disease burden is great, the vaccine-related risk is low, prevention in this setting is more feasible than treatment, the vaccine duration is probably sufficient for the vulnerable period of the child’s life, cost is reasonable, and herd immunity is possible [60].

### Limitations

As with any modeling exercise, the necessary simplification of a complex reality implies limitations that must be considered in applying the results. Since there is almost no surveillance data to specify the etiology of diarrhea during a humanitarian emergency, we had to estimate the rotavirus prevalence. If the true disease incidence is different than the estimated disease incidence, then the vaccine may be more or less cost-effective. Studies to better assess rotavirus disease burden during a humanitarian emergency would be beneficial in providing a more representative picture of the disease incidence. While we performed sensitivity analysis on the two-week diarrheal disease burden we assumed the proportion that would be severe, moderate, and mild would not change and this may not be reflective of the true proportions.

Post-implementation evaluation and continued surveillance for rotavirus disease will remain critical to define vaccine impact on disease burden. Furthermore, only a one-year analytic horizon was examined, which did not take into consideration the additional benefit for the vaccinated children beyond their first year of life. However, one study found that only 18% of rotavirus infections occurred after one year of age [29]. We did not take into account the effects of herd immunity of rotavirus vaccination, which may lower disease burden and further increase benefit of vaccination.

The model also did not consider possible vaccine side effects, including intussusception that might become apparent with large-scale implementation of the vaccine. Post-marketing surveillance has identified a small risk of intussusception associated with the rotavirus vaccine [61,62]. However, given the probable high burden of rotavirus disease in humanitarian emergencies, it would be very unlikely that this risk would significantly alter the cost-effectiveness of the vaccine. In addition, Patel, *et al*. have argued for the removal of the age restriction of vaccination as they showed the benefit of vaccination to age three years overweighed the risk of deaths associated with intussusception [28]. A different distribution of healthcare seeking behavior than that used in our models will influence the economic burden and cost-effectiveness of the vaccination campaign. The range of vaccine coverage was an estimate and may not be a true estimate that can be achieved in Somalia during RI or a SIA.

## Conclusions

The ability to illustrate the cost-effectiveness of a rotavirus vaccination campaign during a humanitarian emergency adds another layer of evidence that such programs represent good investments. The number of humanitarian emergencies has been on the rise, leading to a record number of people, particularly children aged under one year, at risk for infectious diseases [63]. Diarrhea remains a leading cause of mortality among children in low-income countries; many of these countries are at risk for experiencing or have experienced a humanitarian emergency. The reduction of child mortality is one of the United Nations’ Millennium Development Goals, decreasing child mortality during a humanitarian emergency will aid in reaching this goal. The decision to implement vaccination against a high-risk disease during an emergency should be made on the basis of epidemiological, vaccine, political, logistical, and ethical consideration that are specific to the context of the emergency [26]. By showing that rotavirus vaccination serves to reduce morbidity and mortality and is cost-effective, may help augment interventions during a humanitarian emergency.

## Abbreviations

SIA: Supplementary immunization activity
RI: Routine immunization
ICER: Incremental cost-effectiveness ratio
DALY: Disability-adjusted-life-years
WHO: World Health Organization
MICS: Multiple indicator cluster survey
